# Advances and applications of engineered protein therapeutics and their role in precision medicine

**DOI:** 10.3389/fbioe.2026.1857362

**Published:** 2026-10-05

**Authors:** Wadha Aldossary, Nada Alqahtani, Layla Asiri, Noora Alkhurayef, Samar Alagl, Sana Alturki, Duha Saad, Mashael Al Yahya, Samah Al-Harthi

**Affiliations:** Saudi Food and Drug Authority, Riyadh, Saudi Arabia

**Keywords:** biologics, directed evolution, engineered protein therapeutics, monoclonal antibodies, precision medicine, protein engineering, recombinant DNA technology

## Abstract

Proteins are a vital class of biomolecules. They play essential roles in biological processes, including building cellular structures, forming cellular channels and receptors, catalyzing biological reactions, transporting molecules, and contributing to immune defense mechanisms. Advances in protein engineering have enabled the genetic modification of naturally occurring proteins, using recombinant deoxyribonucleic acid (DNA) technology, leading to the development of therapeutic proteins with enhanced specificity, stability, and clinical performance. Despite the rapid growth of engineered biologics, a comprehensive understanding of how these innovations intersect with precision medicine remains limited. This review addresses this gap by summarizing the major contemporary approaches used in therapeutic protein engineering, including directed evolution, rational design, and *de novo* protein design, and by analyzing their roles in enabling patient-tailored interventions. It provides an integrated overview of the clinical applications of engineered therapeutic proteins across key medical fields, supported by a list of U.S. Food and Drug Administration- and European Medicines Agency-approved protein therapeutics from 2020 to 2025. The review further provides a comparative perspective on major protein engineering strategies, highlighting their relative advantages and limitations, while examining differences in clinical translation across major disease areas. By combining technological advances with disease-specific applications, this review offers a perspective that has not been fully developed in the literature. Finally, the review discusses the current challenges affecting the implementation of precision medicine, including immunogenicity, regulatory constraints, and ethical considerations, and outlines future directions, such as artificial intelligence-guided protein design, targeted protein degradation, and personalized vaccine development. Collectively, these insights highlight the expanding role of engineered proteins in shaping the next-generation of precision therapeutics and emphasize the growing importance of integrating multidisciplinary approaches to advance therapeutic innovation in modern clinical practice.

## 1 Introduction

Protein-based therapeutics are a class of biotherapeutics recognized as viable alternatives to traditionally used small-molecule drugs because of their high specificity, low toxicity, and favorable solubility (Carter, 2011). Such therapeutics, including monoclonal antibodies (mAbs), hormones, enzymes, growth factors, clotting factors, and antibody-drug conjugates (ADCs), are produced by modifying native or engineered proteins using biotechnology to enhance or alter their functional characteristics for the treatment of various medical conditions (Dimitrov, 2012; Ebrahimi and Samanta, 2023). Modified protein-based therapeutics are generated by post-translational modifications (PTMs) of naturally occurring proteins through chemical or enzymatic processes to expand the diversity of protein biological functions via the covalent addition of functional groups, proteolytic cleavage, or protein degradation (Lee et al., 2023). The most common PTMs include phosphorylation, glycosylation, ubiquitination, methylation, and acetylation, which generate proteins with enhanced solubility, activity, stability, prolonged circulation time, unique functions, and improved immunogenicity profiles for diverse therapeutic applications, thereby improving their efficacy and pharmacokinetic properties (Lee et al., 2023; Meng et al., 2021). In contrast, engineered protein-based therapeutics, such as Fc-fusion proteins, ADCs, and bispecific mAbs, are naturally occurring proteins that have been genetically modified using recombinant DNA technology to alter their structure and function or to give them novel properties (Balabanova et al., 2015). These approaches range from introducing specific modifications, such as point mutations, domain swapping, and scaffold redesign, to generating and screening large libraries of semi-random polypeptides or creating multispecific constructs, such as bispecific antibodies and fusion proteins, to achieve new or enhanced therapeutic functions (Balabanova et al., 2015; Ebrahimi and Samanta, 2023). The primary aim of protein engineering is to modify binding specificity, enhance affinity, reduce off-target effects, or develop entirely novel biological mechanisms that do not exist in nature, thereby providing a framework for personalized treatments and addressing unmet medical needs (Ebrahimi and Samanta, 2023). Both approaches are essential for improving the efficacy, safety, and versatility of protein-based therapies.

The number of approved protein-based therapeutics has increased dramatically since the introduction of the first U.S. Food and Drug Administration (FDA)-approved recombinant protein therapeutic, Humulin (a brand name for human insulin), in 1982 (U.S. Food and Drug Administration (FDA), 2026a). Protein-based therapeutics are among the fastest-growing segments of the pharmaceutical industry, representing a market of approximately $400 billion, with numerous products approved or currently undergoing clinical trials (Chung et al., 2021). Over the past 5 years, the FDA has approved 213 drugs, including 44 mAbs, which represented approximately 20.7% of these approvals. mAbs also represent a substantial proportion of the best-selling biologic products worldwide (Research and Markets, 2022). The continued approval of therapeutics for oncology and autoimmune diseases has been a major driver of the market dominance of these agents. Examples include trastuzumab, bevacizumab, adalimumab, and rituximab (Siddiqui, 2025). Other recently approved protein therapeutics include Adstiladrin, approved on 1 May 2024, for high-risk non-muscle-invasive bladder cancer, and Abecma, approved on 4 April 2024, for multiple myeloma (U.S. Food and Drug Administration (FDA), 2026b). Ogluo was approved on 11 February 2021, for the treatment of severe hypoglycemia in children aged 2 years and older with diabetes (European Medicines Agency (EMA), 2026h). These examples highlight the significance and diversity of therapeutic proteins in clinical practice.

Therapeutic proteins have been classified using several complementary approaches. Such classification is important for defining the functions of therapeutic proteins within the human body, identifying their targets, and describing their mechanisms of action in the treatment of specific diseases. A key classification proposed by Leader et al. (2008) is based on therapeutic function and clinical application. Another classification groups therapeutic proteins into five categories according to their pharmacological activity (Dimitrov, 2012; Naz et al., 2022) (Table 1). A third classification is based on molecular type and includes antibody-based drugs, anticoagulants, blood factors, bone morphogenetic proteins, engineered protein scaffolds, enzymes, Fc-fusion proteins, growth factors, hormones, interferons, interleukins, and thrombolytics. Proteins can also be classified into three categories according to their molecular mechanisms: (a) specific noncovalent binders, (b) proteins affecting covalent bonds, and (c) proteins that exert activity without specific interactions (Dimitrov, 2012). Another classification focuses on molecular mechanisms and distinguishes proteins that bind noncovalently from those that influence covalent interactions (Pisal et al., 2010). These classification frameworks provide researchers and clinicians with a foundation for understanding, developing, and applying protein therapeutics.

**TABLE 1:** Illustrative classification of therapeutic proteins based on function, therapeutic application, and pharmacological activity.

Classification	Group	Description	Examples
Function and therapeutic application (Leader et al., 2008)	(I) Enzymatic or regulatory activity	This group can be further classified into three categories Ia: Treatment of deficient or abnormal proteins, including hormones and metabolic proteins. Ib: Enhancement of specific pathways, including hormones, blood proteins, and immune response proteins. Ic: Generation of new functions from naturally occurring proteins to modify physiological processes associated with disease.	Ia: Humulin, Novolin Ib: Epogen, Activase Ic: Botox, Refludan
	(II) Target-specific proteins	This group can be further classified into two categories IIa: Binding to molecules or organisms to block their function or enhance specific signaling pathways. IIb: Delivery of specific compounds to target sites.	IIa: Humira, Zenapax IIb: Ontak, Zevalin
	(III) Vaccines	Recombinant vaccines are classified into three subgroups IIIa: Protection against foreign substances. IIIb: Applications in autoimmune disorders. IIIc: Applications against cancer.	Engerix, Recombivax HB
	(IV) Diagnostics	Recombinant proteins used for diagnostic purposes, including infectious disease diagnostics and imaging agents for cancer detection (Akash et al., 2015).	GlucaGen, Verluma
Pharmacological activity (Dimitrov, 2012; Naz et al., 2022; EMA, 2026d; EMA, 2026i)	Replacing a deficient or abnormal protein	Replacement of deficient or abnormal proteins.	Advate, Novolin
	Increasing an existing pathway	Modification of signaling pathways.	Aldesleukin, Darbepoetin alfa
	Proteins with novel functions or activities	Proteins that exhibit novel or enhanced biological activities.	Hemlibra, Blincyto
	Interfering with molecules or organisms	Proteins that interact with specific molecules or target foreign antigens, including viruses and bacteria.	Enbrel, Peginterferon alfa-2a
	Transporting other compounds or proteins	Proteins that function as carriers for therapeutic compounds or proteins.	Elaprase, Abraxane

Owing to advances in therapeutic protein development, precision medicine has emerged as a transformative approach to healthcare. Precision medicine involves tailoring treatments to an individual patient or a group of patients according to their unique characteristics. Integrating therapeutic proteins into precision medicine relies on stratifying patients according to their genetic, environmental, and behavioral characteristics to improve therapeutic outcomes (Ginsburg and Phillips, 2018). One example is trastuzumab, a mAb used to treat patients with human epidermal growth factor receptor 2 (HER2)-positive breast cancer by selectively targeting and binding to the overexpressed HER2 receptor (Slamon et al., 2001). This concept is closely associated with next-generation protein therapeutics, which are advanced treatments designed to overcome the limitations of earlier protein therapeutics while enhancing specificity and biological activity. Examples include bispecific antibodies, ADCs, fusion proteins, and engineered cytokines (Beck et al., 2017; Leader et al., 2008).

Integrating precision medicine with next-generation protein therapeutics has marked a major advance by enabling the selective targeting of diseases based on individual patient characteristics. For example, bispecific T-cell engagers (BiTEs) direct a patient’s immune cells to selectively bind tumor cells expressing specific antigens (Kontermann, 2012). These targeted approaches improve clinical outcomes while reducing off-target effects. Building on such approaches, precision medicine should focus on developing dynamic treatment regimens and personalized strategies that can be adapted to patient-specific characteristics. Its emphasis on data-driven, patient-centered care, together with challenges such as patient heterogeneity, implementation costs, and causal complexity, establishes precision medicine as a distinct field of study (Kosorok and Laber, 2019). In this context, disease modeling supports the understanding of disease mechanisms and the development of effective treatment strategies.

This review outlines the fundamental techniques used in protein engineering and discusses the applications of therapeutic proteins across different medical fields. It highlights emerging research directions, including how engineered proteins are being integrated into precision medicine to enable patient-specific therapies. The review concludes by examining the current challenges and limitations of engineered protein development, together with future opportunities that may shape innovations in targeted and personalized treatments.

Although previous studies have focused primarily on protein characterization and the general mechanisms of therapeutic proteins, this review addresses an important gap by bringing together manufacturing, regulatory, and ethical considerations related to protein therapeutics used in precision medicine. It provides an overview of the major protein engineering techniques and recent advances across key therapeutic areas, including oncology, hematology, infectious diseases, and other clinically relevant fields. It also discusses how these engineered protein modalities can support precision medicine by enabling patient stratification and personalized treatment strategies. In addition, this review summarizes FDA- and European Medicines Agency (EMA)-approved engineered protein therapeutics from the past 5 years, providing an up-to-date reference that links recent regulatory trends with emerging scientific and clinical applications.

## 2 Methods

This narrative review provides a comprehensive overview of recent advances in engineered protein therapeutics and their emerging roles in precision medicine. It focuses on the key techniques, recent applications, and future directions of protein therapeutics across different fields. Literature selection was guided by relevance, significance, and recency rather than by a predefined systematic protocol. To identify key research articles, a comprehensive literature search was conducted using PubMed, Scopus, Web of Science, and Google Scholar with the following keywords: Protein Therapeutics, Precision Medicine, Personalized Medicine, Recombinant Proteins, Biosimilars, Drug Targeting, Fusion Proteins, Monoclonal Antibodies, Directed Evolution, Rational Design, *De Novo* Protein Design, and Synthetic Biology. The search included articles published between 2015 and 2025, with an emphasis on the last 5 years to reflect the rapid technological evolution of protein engineering and its convergence with personalized therapeutic approaches. Articles were included based on their relevance to the central themes of this review, including protein engineering approaches, recent advances in the design and engineering of therapeutic proteins, and their therapeutic applications in precision medicine.

## 3 Comparative analysis and methodological trade-offs in protein engineering

Rapid advances in the biological sciences, particularly in recombinant DNA technology, have led to the development of various protein engineering approaches. Three primary strategies are used in protein engineering: directed evolution, rational design, and *de novo* protein design (Table 2; Figure 1) (Dauparas et al., 2022; Pan and Kortemme, 2021). In addition to these three approaches, other strategies include synthetic biology and protein delivery systems. These techniques have been used to generate new proteins or improve the properties of existing proteins for applications in biotechnology and medicine. The choice of protein engineering approach depends on the balance between experimental throughput and targeted design. These strategies are not independent alternatives; each involves analytical and methodological trade-offs in cost, processing time, and practical constraints.

**TABLE 2:** Comparison of directed evolution, rational design, and *de novo* protein design approaches.

Aspect	Directed evolution	Rational design	*De novo protein design*
Definition	Mimics natural selection to evolve proteins toward desired traits.	Creates desired alterations in proteins by directly modifying amino acids (Reetz et al., 2023; Satapathy et al., 2022).	Generates new sequences predicted by AI models to fold into desired structures, enabling the creation of entirely new proteins (Pan and Kortemme, 2021).
Methodology	Mutagenesis and selection/screening approaches, including error-prone PCR (epPCR), homologous recombination, and phenotypic assays (Wang X et al., 2021).	Site-directed mutagenesis and computational modeling (Reetz et al., 2023).	Machine learning and structure modeling (Pan and Kortemme, 2021).
Time efficiency	Provides results rapidly, often within a few weeks.	Less time-consuming because it does not require large-scale library screening (Liu et al., 2022; Reetz et al., 2023).	Requires a longer development period, often several months or more.
Knowledge requirement	Requires knowledge of molecular biology, mutagenesis techniques, selection and screening methods, and bioinformatics tools (Wang Y et al., 2021).	Requires prior knowledge of the target protein’s structure–function relationship, site-directed mutagenesis techniques, and bioinformatics tools (Liu et al., 2022).	Requires AI and machine learning expertise (Kortemme, 2024).
Tools	Genetic diversity generation (Pan and Kortemme, 2021) Error-prone PCR DNA shuffling (Pan and Kortemme, 2021) display systems (Dauparas et al., 2022; Wang et al., 2020) Screening and selection High-throughput screening (HTS) (Ren et al., 2019; Ye et al., 2020) Fluorescence-activated cell sorting (FACS) (Hendel and Shoulders, 2021; Sellés Vidal et al., 2023) Biosensors Transcription factor-based biosensors (Ren et al., 2019; Snoek et al., 2020). Fluorescent biosensors (Kuldyushev, 2025; Liu et al., 2022) Computational tools and machine learning In silico simulation tools (Anishchenko et al., 2021)	Structure determination X-ray crystallography, Nuclear magnetic resonance (NMR) spectroscopy, Cryo-electron microscopy (Goverde et al., 2023; Reetz, 2022) Computer-aided designs (*in silico*) (Reetz, 2022) Protein structure prediction: alpha Fold, PyMOL, ChimeraX, Mutation impact: FoldX, I-mutatant 3.0, mCSM, Site-directed mutagenesis (SDM). Docking and molecular dynamics: AutoDock, ClusPro, NAMD, GROMACS. AI and deep learning (DL): Rosetta, alpha fold Goverde et al. (2023) and Reetz (2022)	Machine learning models AlphaFold (Goverde et al., 2023; Pan and Kortemme, 2021), Rosetta (Pan and Kortemme, 2021), ProteinMPNN (Dauparas et al., 2022).
Application	Biocatalysis, therapeutics, synthetic biology strain improvement, development of stable enzymes (Sellés Vidal et al., 2023), antibodies (Sellés Vidal et al., 2023), and diagnostics (Sandomenico et al., 2020).	Nanomedicine (Shan et al., 2024), industrial biocatalysis (Ndochinwa et al., 2024), and vaccine development (Vishweshwaraiah and Dokholyan, 2022).	Enzymes, therapeutic proteins (Vanella et al., 2022), diagnostics (Dauparas et al., 2022), and bioengineering applications (Pan and Kortemme, 2021; Vanella et al., 2022).
Example of use	Development of thermostable enzymes for industrial applications (Ndochinwa et al., 2024; Sellés Vidal et al., 2023). Mutated genes in *B. subtilis* metabolic variants (Sellés Vidal et al., 2023).	Improvement of enzyme thermostability (Pongsupasa et al., 2022; Zhou and Wang, 2021) Development of peptide-based inhibitors to disrupt protein–protein interactions (Wang et al., 2021) Peptide-based smart hydrogels for therapeutic applications (Das and Das, 2021) Allosteric modulators (Chatzigoulas and Cournia, 2021) Development of new antibiotic classes for drug-resistant infections (Durand-Reville et al., 2021)	Redesigned monomers and nanoparticles (Dauparas et al., 2022). https//pubmed.ncbi.nlm.nih.gov/36108050/IL-2 mimetic protein for cancer immunotherapy (Pan and Kortemme, 2021).
Advantages	Fast, versatile, and does not require prior structural knowledge (Sellés Vidal et al., 2023).	Increases the likelihood of obtaining advantageous mutations (Zhou and Wang, 2021).	Higher sequence recovery than traditional methods (Dauparas et al., 2022; Pan and Kortemme, 2021).
Limitations	Screening bottlenecks and limited structural understanding (Sellés Vidal et al., 2023).	Requires accurate structural modeling and optimization of intermolecular interactions and protein stability.	Challenging to reliably design proteins with novel functions (Dauparas et al., 2022).

**FIGURE 1:**
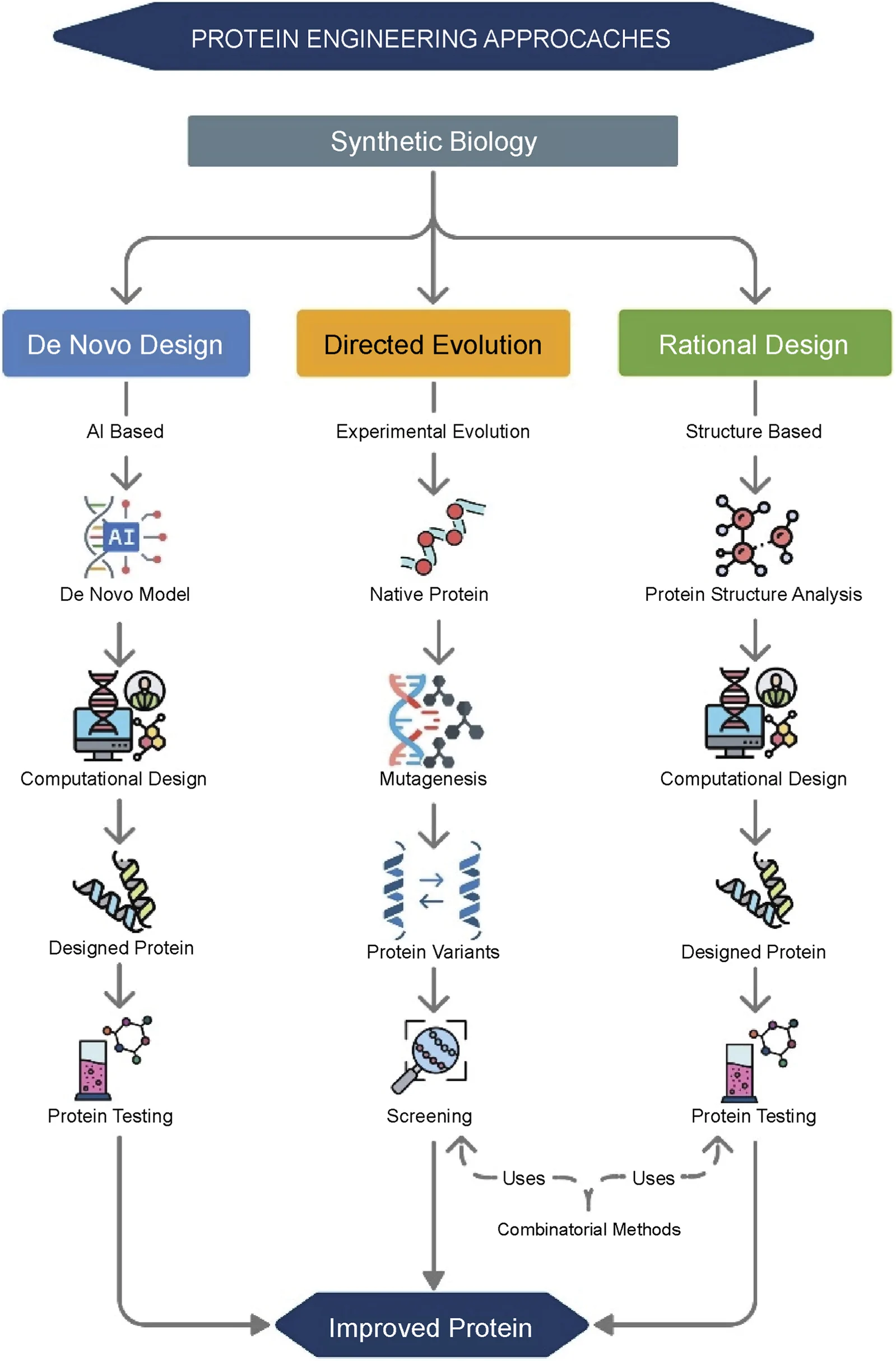
This diagram illustrates the main strategies used in protein engineering to modify existing proteins or create new proteins with improved or novel functions. *De novo* design creates new proteins from scratch, often using advanced computational tools and AI to identify unique structures and functions. Directed evolution mimics natural selection by improving existing proteins through iterative cycles of random mutagenesis followed by selecting or screening variants with desirable properties. This approach relies heavily on combinatorial methods to generate and evaluate large libraries of protein variants. In contrast, rational design modifies proteins based on a detailed understanding of their three-dimensional (3D) structure and its relationship to function. Finally, synthetic biology, a broader interdisciplinary field, incorporates protein-engineering strategies as key tools for designing and constructing complex biological systems and pathways with specific engineered functions.

### 3.1 Engineering sequence diversity: from directed evolution to structure-based design

Directed evolution mimics natural selection to evolve proteins or nucleic acids toward user-defined traits through iterative rounds of mutation and selection (Wang et al., 2020). The process begins with genetic diversification using techniques such as error-prone polymerase chain reaction (PCR), DNA shuffling, or site-directed mutagenesis to generate a library of variants (Figure 1) (McLure et al., 2022). Selection and screening methods, such as phage display, yeast surface display, and high-throughput assays, then identify the variants with enhanced or novel properties (Sellés Vidal et al., 2023; Ye et al., 2020). Key methods include fluorescence-activated cell sorting (FACS), ribosome display, biosensors, and various mutagenesis strategies.

Unlike directed evolution, which screens large libraries of random variants, rational design is a classical protein engineering approach based on targeted, structure-guided substitution. It rests on predicting how a molecule’s structure, inferred primarily from its motifs, influences its behavior. Researchers then modify or introduce selected amino acids or domains into the polypeptide backbone to generate proteins with new or altered biological functions (Figure 1). Rational design combines experimental and computational tools to improve analytical accuracy, guide design choices, and predict protein behavior. X-ray crystallography and related techniques determine the three-dimensional structures of proteins. Computational tools such as FoldX and I-Mutant assess the effects of mutations on protein stability and function, whereas AutoDock performs molecular docking simulations. Rational design workflows increasingly incorporate artificial intelligence (AI) and deep learning platforms, such as Rosetta, to improve prediction accuracy and streamline docking.

The most innovative of these approaches, *de novo* protein design, builds protein sequences and structures that do not exist in nature. This approach uses fundamental physical principles and advanced computational models to predict how amino acid sequences fold into specific three-dimensional structures, enabling the design of proteins with tailored functions (Figure 1) (Anishchenko et al., 2021; Pan and Kortemme, 2021). The principal tools include Rosetta, AlphaFold, and ProteinMPNN (Dauparas et al., 2022). Rosetta applies physics-based algorithms to model macromolecular interactions and guide sequence optimization, whereas AlphaFold, developed by DeepMind, uses deep learning to predict protein structures with remarkable accuracy (Anishchenko et al., 2021; Goverde et al., 2023). ProteinMPNN, a more recent advancement, uses neural networks to design sequences compatible with a specified protein backbone. Compared with previous methods, it achieves higher sequence recovery and better functional performance (Dauparas et al., 2022).

Although directed evolution does not require detailed structural knowledge, it can be resource-intensive and may be limited by screening bottlenecks. Nevertheless, it offers a rapid development cycle and broad applicability, making it an essential technique in modern protein engineering (Ren et al., 2019; Ye et al., 2020). Unlike directed evolution, rational design requires a detailed understanding of the structural, functional, and molecular properties of proteins. However, AI has substantially improved protein structure prediction based on amino acid sequences (Liu et al., 2022). For *de novo* design, challenges remain in reliably engineering proteins with entirely new functions, highlighting the need for continued research and methodological refinement (Anishchenko et al., 2021; Dauparas et al., 2022). These differences shape where each approach is applied. Directed evolution is widely used in industrial biocatalysis, pharmaceuticals, synthetic biology, and environmental biotechnology. For example, enzymes have been evolved to remain active under extreme thermal conditions, thereby improving their utility in industrial applications (Ren et al., 2019). Rational design has been applied to develop protein-based vaccines, antibodies, and enzymes with enhanced thermal stability and catalytic efficiency to meet industrial demands. In contrast, *de novo* protein design has been used to develop novel enzymes, therapeutic proteins, and molecular tools with specific binding affinities. Accordingly, rational design is generally preferred when sufficient structural and mechanistic information is available, directed evolution is advantageous when such knowledge is limited and experimental screening is feasible, whereas *de novo* design is most suitable when entirely new protein structures or functions are required.

### 3.2 Other strategies: synthetic biology

Synthetic biology is an interdisciplinary field based on engineering principles. It involves the design, construction, and modification of biological systems, including organisms, pathways, and biological devices, using engineering tools and techniques to achieve specific practical goals (Xin et al., 2019). The process begins with designing a biological system and identifying its required components using advanced software that simulates system behavior. The biological components are then constructed using techniques such as DNA synthesis and gene-editing technologies. These components are subsequently introduced into a host organism to establish the desired biological function. The resulting system is then tested experimentally by collecting data, comparing the observed outcomes with the predicted model, and analyzing any discrepancies. This information is used to refine the design, and these iterative design-build-test-learn cycles are repeated until the desired biological performance is achieved (Garner, 2021). Scientists and biotechnology companies worldwide use synthetic biology to address challenges in manufacturing, agriculture, and medicine. Its applications are broad and include improving diagnostics, developing targeted therapies, and creating drugs with fewer adverse effects (Garner, 2021). Protein engineering has also evolved alongside advances in machine learning, proteomics, metabolomics, and genomics. By integrating multi-omics data, researchers can generate precise protein variants and identify novel enzymes for natural product biosynthesis. These advances, driven by synthetic biology, have contributed substantially to public health by enabling the development of next-generation therapies for infectious diseases and cancer (McCarty and Ledesma-Amaro, 2019).

Although directed evolution, rational design, and *de novo* protein design are generally considered independent strategies, integrating them is an important advance in biotechnology. Whereas directed evolution relies extensively on laboratory experimentation, rational design depends on structural analysis of proteins. Computational tools can be used to refine protein designs before experimental validation, thereby reducing the resources required for traditional experimental approaches. Future developments in the field should extend beyond static protein structure prediction toward a better understanding of the dynamic functional behavior of therapeutic proteins within biological systems.

## 4 Integrating computational frameworks and bridging the methodological gap

AI is now reshaping protein design strategies and provides a robust framework for bridging the methodological gap in protein engineering. As described by Notin et al. (2024), machine learning models have become the cornerstone of this approach by overcoming the limitations of traditional evolutionary approaches. Notin et al. (2024) categorized machine learning models based on protein sequence, structure, and functional annotations, providing a clear framework that bridges structural bioinformatics and practical protein engineering applications. Moreover, recent advances in deep learning platforms, such as AlphaFold and Rosetta, have transformed rational design into a precision-oriented predictive approach. In addition, integrating molecular dynamics simulations with structural bioinformatics has improved the accuracy of characterization and evaluation of complex protein structures. Separately, AlphaFold2 has been used to predict protein structures and analyze protein variants (Sajeev-Sheeja et al., 2025). Collectively, these computational approaches are driving the development of next-generation engineered proteins for precision medicine.

## 5 Advances in protein delivery systems: strategies to improve pharmacological properties and delivery

### 5.1 Targeted delivery mechanisms

Targeted delivery systems are designed to carry drugs to specific sites rather than throughout the entire body or organ, and they integrate principles from fields such as polymer science and molecular biology (Tewabe et al., 2021). These strategies enable the precise and efficient delivery of therapeutic agents to tumor cells or tissues while minimizing exposure to healthy cells and tissues (Gao et al., 2023).

Nanotechnology offers an effective approach to drug delivery, particularly through nanoparticles that target tumors via the enhanced permeability and retention effect (Dang and Guan, 2020). Recent strategies have combined targeting molecules, such as antibodies and peptides, with delivery carriers, including liposomes and polymers (Chamundeeswari et al., 2018). Liposomes provide controlled drug release, reduced toxicity, and protection of therapeutic agents at target sites (Antimisiaris et al., 2021). In addition, tumor-targeting peptides bind to specific receptors, thereby enhancing the antitumor activity of nanoparticle-loaded drugs (Lamb et al., 2024; Li et al., 2023). Several studies have also reviewed the applications of cell-penetrating peptides in cancer therapy (Lamb et al., 2024; Moreno-Vargas and Prada-Gracia, 2024).

Designing and optimizing these targeting molecules and delivery vehicles strengthens interactions with tumor cells and improves drug delivery efficiency. Furthermore, nanocarrier-based targeted drug delivery increases drug accumulation in tumor tissues while reducing drug distribution to healthy tissues (Kothari et al., 2024).

### 5.2 Stabilization and half-life enhancement

PEGylation involves the covalent attachment of polyethylene glycol (PEG), a non-toxic, flexible, and water-soluble polymer, to peptides or proteins. Studies have shown that PEG conjugation substantially prolongs circulation time, reduces immunogenicity, enhances solubility, and improves the physical stability of therapeutic agents (Li et al., 2024; Zaman et al., 2019). PEG is one of the most widely used polymers for drug delivery because of these favorable properties and its neutral charge. Its major advantages include improving the stability of conjugated proteins and protecting therapeutic agents from immune-mediated degradation by reducing immunogenicity (Binder and Skerra, 2025; Zaman et al., 2019).

Several additional strategies have recently been developed to extend the half-life of therapeutic proteins, including amino acid modifications, genetic fusion with serum proteins, and polymer conjugation such as PEGylation and polysialylation (Binder and Skerra, 2025; Zaman et al., 2019). The two most commonly used approaches are reducing renal clearance and exploiting the neonatal Fc receptor (FcRn)-mediated recycling pathway through binding to albumin or the Fc region of immunoglobulin G (Foss et al., 2024; Ko et al., 2025; Tan et al., 2018).

## 6 Protein-engineering applications and their role in precision medicine

### 6.1 Genetic disorders

Genetic disorders arise from alterations in DNA sequences, including single-gene mutations (monogenic disorders), chromosomal abnormalities, and mitochondrial defects (National Human Genome Research Institute, 2026; Ziats et al., 2020). They manifest clinically as developmental delay, metabolic dysfunction, neuromuscular degeneration, hematologic disorders, and immunodeficiencies (Ziats et al., 2020). Duchenne muscular dystrophy, spinal muscular atrophy (SMA), cystic fibrosis, hemophilia, and phenylketonuria are well-known examples. Historically, treatment was largely limited to symptom management because the underlying genetic causes could not be effectively targeted (Ziats et al., 2020). Advances in protein engineering have expanded treatment options by enabling the development of therapeutic proteins that replace deficient or dysfunctional proteins, restore specific biological functions, or modify disease-associated pathways. Such approaches are particularly relevant to monogenic disorders in which the underlying molecular defect and its functional consequences are well characterized. The increasing ability to tailor therapeutic design to specific molecular abnormalities has therefore strengthened the role of engineered proteins in the precision treatment of genetic diseases.

Several biologics targeting genetic disorders received FDA and EMA approval between 2020 and 2025 (Supplementary Table S1). More recently, precision medicine has shifted this approach toward mechanism-based therapeutics (Diseases | GARD, n. d.; “Notable approvals | Drugs | U.S. Food and Drug Administration (FDA), 2026c; European Medicines Agency (EMA), 2026f; Fatima et al., 2023; U.S. Food and Drug Administration (FDA), 2026h). Table 3 summarizes engineered therapeutic approaches for these conditions.

**TABLE 3:** Engineered therapeutic proteins across multiple therapeutic fields.

Field	Protein-engineering approach	Mechanism explanation	Drug example	Application/condition
Oncology	Engineered cell therapies (CAR-T cell)	Engineered cell therapies, including CAR-T, TCR-T, and TIL therapies, are developed through viral transduction of a patient’s own immune cells (T cells), which are modified to recognize and attack cancer cells.	Abecma idecabtagene vicleucel (U.S. Food and Drug Administration (FDA), 2026b)	Multiple myeloma.
	Bispecific T-cell engager (BiTE)	Dual scFv targeting of BCMA and CD3.	Tecvayli teclistamab (U.S. Food and Drug Administration (FDA), 2026g)	Relapsed/refractory multiple myeloma.
	Monoclonal antibodies (mAbs)	Bind to the PD-1 receptor and block its interaction with PD-L1 and PD-L2	Dostarlimab (U.S. Food and Drug Administration (FDA), 2026d)	Endometrial cancer.
	Immune checkpoint inhibitors (ICIs) and antibody combinations	Dual checkpoint inhibition (PD-1 + LAG-3) to reverse T-cell exhaustion.	Opdualag nivolumab + relatlimab (U.S. Food and Drug Administration (FDA), 2026f)	Unresectable/metastatic melanoma.
	Antibody-drug conjugates (ADCs)	Anti-folate receptor alpha antibody linked to a maytansinoid payload.	Elahere mirvetuximab soravtansine (U.S. Food and Drug Administration (FDA), 2026e)	Platinum-resistant ovarian cancer.
Genetic disorders	Antibody fragments (scFv)	Enhanced antigen specificity with reduced molecular size.	Anti-vascular endothelial growth factor (VEGF) therapy (e.g., brolucizumab) (Garrelfs et al., 2021; Zhang et al., 2022)	Inhibition of VEGF-A in macular degeneration.
	RNA-based therapeutics (small interfering RNA siRNA)	Gene silencing through degradation of pathogenic mRNA.	Lumasiran (Oxlumo) (Garrelfs et al., 2021)	Primary hyperoxaluria type 1
	CRISPR/Cas9 gene editing	Reactivation of fetal hemoglobin via gene disruption.	Exagamglogene autotemcel (Casgevy) (Hoy, 2024; U.S. Food and Drug Administration (FDA), 2023b)	Sickle cell disease
	Recombinant enzyme replacement therapy	Glyco-optimized enzyme designed to improve lysosomal uptake.	Avalglucosidase alfa (Kishnani et al., 2023)	Pompe disease
	Peptide analog	Modulation of the fibroblast growth factor receptor 3 (FGFR3) pathway to promote bone growth.	Vosoritide (Sävendahl et al., 2020)	Achondroplasia
	Adeno-associated virus (AAV)-based gene replacement	Delivery of functional genes to restore protein expression.	Etranacogene dezaparvovec (HemGenix) (European Medicines Agency (EMA), 2026c)	Hemophilia B
	*Ex vivo* gene-modified cell therapy	Insertion of the β-globin gene into patient-derived stem cells.	Betibeglogene autotemcel (Locatelli et al., 2022)	β-thalassemia
Endocrinology and metabolism	scFv-peptide fusion	Glucagon-like peptide-1 receptor (GLP-1R) agonist activity with improved targeting.	GLP-1R fusion (e.g., mGLP1 DARPin) (Ehsasatvatan and Baghban Kohnehrouz, 2023)	Type 2 diabetes mellitus
	Allogeneic cell therapy	Restoration of insulin production via islet transplantation.	Zimislecel (U.S. Food and Drug Administration (FDA), 2023a; Reichman et al., 2025)	Type 1 diabetes mellitus
	Long-acting hormone analog	Albumin binding to extend the half-life of growth hormone (GH).	Somapacitan (Simons et al., 2023)	Growth hormone deficiency
	Recombinant lysosomal enzyme	Degradation of accumulated oligosaccharides.	Velmanase alfa (Kishnani et al., 2023; EMA, 2026b)	Alpha-mannosidosis
Bridging genetic and metabolic disorders	Recombinant enzyme replacement	Glycosylation-enhanced acid alpha-glucosidase (glyco-optimized GAA) improves lysosomal uptake.	Avalglucosidase alfa (Kishnani et al., 2023)	Pompe disease
	AAV2-based gene therapy	Restores aromatic L-amino acid decarboxylase (AADC) enzyme expression in the central nervous system (CNS).	Eladocagene exuparvovec (Wang et al., 2021)	AADC deficiency
	MC4R agonist	Activates appetite-suppressing pathway.	Setmelanotide (Clément et al., 2020; Kühnen et al., 2016)	Genetic obesity (proopiomelanocortin [POMC] and leptin receptor [LEPR] deficiencies)
	RNA-based therapeutics (siRNA)	Suppresses 5-aminolevulinate synthase 1 (ALAS1) to reduce the accumulation of toxic heme intermediates.	Givosiran (Balwani et al., 2020)	Acute hepatic porphyria
	RNA-based therapeutics (siRNA)	Inhibits hydroxyacid oxidase 1 (HAO1) to reduce oxalate production.	Lumasiran (Oxlumo) (Garrelfs et al., 2021)	Primary hyperoxaluria type 1
	RNA-based therapeutics (siRNA)	Silences proprotein convertase subtilisin/kexin type 9 (PCSK9) to reduce low-density lipoprotein cholesterol (LDL-C) levels.	Inclisiran (Ray et al., 2020)	Hypercholesterolemia
Autoimmune diseases	Cytokine inhibitors	Cytokines regulate immunity and inflammation. Overproduction of cytokines (e.g., TNF-α, IL-1, IL-6, and IL-31) contributes to chronic inflammation and tissue damage (Diotallevi and Offidani, 2023; Malik and Ghatol, 2023).	Nemolizumab	Prurigo nodularis and Atopic dermatitis (Liang et al., 2022; Raja et al., 2025)
	Immune cell inhibitors	Modulate autoreactive T cells and reduce autoantibody activity, restoring immune balance and delaying disease progression (Wu et al., 2025).	Teplizumab	Type 1 diabetes (delays disease onset) (Buddhavarapu et al., 2024)
	Receptor inhibitors	Neonatal Fc receptors (FcRn) extend IgG lifespan; blocking FcRn reduces circulating pathogenic autoantibodies, including pathogenic auto-IgG (Lamamy et al., 2021).	Efgartigimod alfa	Myasthenia gravis (Heo, 2023)
Neurological disorders	Amyloid-beta targeting	Engineered monoclonal antibodies (mAbs) bind amyloid-beta (Aβ), facilitating its clearance and preventing plaque formation. Accumulation of Aβ and tau aggregates triggers neuroinflammation and neuronal injury (van Dyck et al., 2023).	Lecanemab	Alzheimer’s disease (van Dyck et al., 2023)
​	Complement inhibitors	Bind and inhibit complement components, such as C5 activation. Activation of the complement cascade and formation of the membrane attack complex contribute to destruction of the postsynaptic neuromuscular junction membrane and impaired neuromuscular transmission (Dalakas et al., 2020; Dodd et al., 2026).	Zilucoplan	Myasthenia gravis (Howard et al., 2023)
	CGRP inhibitor	Directly binds and neutralizes human calcitonin gene-related peptide (CGRP), preventing receptor activation and disrupting the CGRP-mediated trigeminal pain pathway (Scheffler et al., 2024). Excessive CGRP activity promotes cranial blood vessel dilation and pain signaling (Hansen and Ashina, 2014).	Eptinezumab	Migraine (Scheffler et al., 2024)
	Nucleic acid-based therapies	Synthetic DNA or RNA sequences modulate gene expression, correct mutations, or replace defective genes, such as the Duchenne muscular dystrophy (DMD) gene. DMD mutations result in the absence of dystrophin, a protein essential for maintaining muscle function (Zaidman et al., 2023).	Delandistrogene moxeparvovec	DMD (Zaidman et al., 2023)
Infectious diseases	Monoclonal antibody engineering (humanized/fully human)	Binds viral antigens (e.g., SARS-CoV-2 spike protein), neutralizes the pathogen, and prevents viral entry into host cells.	Sotrovimab (European Medicines Agency (EMA), 2026k)	COVID-19 treatment
	Fc-fusion protein design	Enhances the half-life and antibody-dependent cellular cytotoxicity (ADCC) activity of neutralizing antibodies.	Palivizumab (European Medicines Agency (EMA), 2026j)	Respiratory syncytial virus (RSV) prophylaxis in high-risk infants
	PEGylation of enzymes	Enhances the stability and circulation time of bacterial-derived enzyme therapies.	Pegademase bovine (Booth and Gaspar, 2009)	Severe combined immunodeficiency (SCID) due to Adenosine deaminase (ADA) deficiency
	Multivalent nanobody engineering	Provides compact, multi-epitope binding to enhance viral neutralization.	Lipoxin A4 receptor ALX-0171 (in trials) (Detalle et al., 2016)	RSV treatment (inhaled nanobody)
	Recombinant interferon optimization	Enhances antiviral immune responses while reducing immunogenicity.	Interferon beta-1a (European Medicines Agency (EMA), 2026m)	Hepatitis B, a viral infection
Hematology	Fc-fusion coagulation factors	Extends half-life through FcRn recycling, reducing dosing frequency.	Efmoroctocog alfa (Mahlangu et al., 2014)	Hemophilia A (factor VIII)
	Glycoengineering of factor IX	Enhances half-life and activity through Fc and glycan modifications.	Eftrenonacog alfa (European Medicines Agency (EMA), 2026a)	Hemophilia B
​	Recombinant erythropoietin analog	Engineered to achieve higher receptor affinity and prolonged half-life.	Darbepoetin alfa (European Medicines Agency (EMA), 2026b)	Anemia associated with chronic kidney disease (CKD) or chemotherapy
	Monoclonal CD20 antibody (chimeric to humanized)	Targets B cells, inducing apoptosis and immune-mediated cell lysis.	Rituximab (European Medicines Agency (EMA), 2026e)	Non-Hodgkin lymphoma and chronic lymphocytic leukemia (CLL)
	Bispecific T-cell engager (BiTE)	Links CD3^+^ T cells with malignant CD19-positive B cells, inducing cytotoxicity.	Blinatumomab (Kantarjian et al., 2017)	B-cell precursor acute lymphoblastic leukemia (ALL)
	Long-acting thrombopoietin receptor agonist	Engineered Fc-peptide fusion stimulates platelet production.	Romiplostim (European Medicines Agency (EMA), 2026g)	Chronic immune thrombocytopenia (ITP)

scFv, single-chain variable fragment; siRNA, small interfering RNA; AAV, adeno-associated virus; GIP, glucose-dependent insulinotropic polypeptide; GLP-1, glucagon-like peptide-1; PCSK9, proprotein convertase subtilisin/kexin type 9.

### 6.2 Endocrine and metabolic disorders

Endocrine and metabolic disorders encompass a wide range of conditions caused by hormonal abnormalities, genetic mutations affecting metabolic pathways, and inherited enzyme deficiencies (Diseases | GARD, n. d.; European Medicines Agency (EMA), 2026f). Examples include diabetes and Pompe disease (Diseases | GARD, n. d.; American Diabetes Association, 2022; European Medicines Agency (EMA), 2026l). Historically, treatment focused primarily on symptom relief through hormone replacement therapy, dietary interventions, and symptom-based pharmacotherapy. Protein engineering has enabled the development of improved hormone analogues, enzyme replacement therapies, and other biologic agents with enhanced stability, pharmacokinetic profiles, and target specificity. These advances are particularly important in disorders where treatment depends on replacing a deficient hormone or enzyme or correcting a specific metabolic pathway. As a result, engineered therapeutic proteins increasingly support more individualized and mechanism-based management of endocrine and metabolic diseases. Several biologics targeting endocrine and metabolic disorders received FDA and EMA approval between 2020 and 2025 (Supplementary Table S1). Table 3 summarizes engineered therapeutic applications in this area.

### 6.3 Oncology

Oncology is at the forefront of modern medicine, addressing one of the greatest challenges in healthcare: cancer, a group of diseases characterized by uncontrolled cell growth and the ability to invade neighboring tissues and affect multiple organs. Conventional cancer treatments are often nonspecific and broadly cytotoxic, resulting in considerable variability in treatment response and adverse effects among patients. Precision medicine has therefore shifted oncology from a one-size-fits-all approach toward biomarker-guided therapies. This approach uses tumor-specific molecular biomarkers and companion diagnostics to guide targeted therapies, as illustrated by HER2-and EGFR-directed treatments.

Despite the promise of precision oncology, several challenges remain. Foremost among these is tumor heterogeneity within and between patients, which complicates biomarker identification and contributes to variable responses to treatment (Dienstmann et al., 2013). In addition, intrinsic, adaptive, and acquired resistance mechanisms limit the long-term efficacy of targeted therapies, necessitating continued innovation in treatment design (Sharma et al., 2017). Safe and effective delivery of cancer biologics also remains difficult. Many biologic agents require highly specific, controlled delivery to tumor tissue while minimizing off-target effects and systemic toxicity. Nanotechnology- and viral vector-based delivery systems are under investigation (Jiang et al., 2023). However, delivery is further complicated by physiological barriers, such as the blood-brain barrier (BBB) in certain cancers and the dense extracellular matrix of solid tumors. Finally, careful oversight is needed for ethical and legal issues such as data privacy, informed consent for genomic testing, and the potential for algorithmic bias in AI-assisted decision support systems (Bruynseels et al., 2018). Addressing these challenges is essential to ensure that precision oncology remains safe, effective, and equitable. Table 3 presents recent advances in targeted protein-based therapies for cancer, including their engineering strategies, mechanisms of action, and therapeutic applications. The FDA and EMA also approved several cancer biologics between 2020 and 2025 (Supplementary Table S1).

### 6.4 Autoimmune diseases

Autoimmune diseases arise from a breakdown of self-tolerance, in which the body’s own tissues come under immune attack (Shirafkan et al., 2024). The resulting conditions range from chronic and progressive to episodic, and include type 1 diabetes, rheumatoid arthritis (RA), and myasthenia gravis. Although the causes often remain unknown, research has clarified the epidemiology, pathogenesis, and triggers of these conditions (Song et al., 2024). Their uncertain etiology and unpredictable clinical manifestations make these diseases difficult to prevent and manage (Yasmeen et al., 2024). Conventional treatment relies primarily on immunosuppressive therapies, which are frequently effective but often leave disease control incomplete and cause systemic adverse effects (Yasmeen et al., 2024). Emerging targeted immunotherapies, including engineered protein therapeutics, offer a more precise treatment approach with reduced toxicity and improved clinical outcomes (Ebrahimi and Samanta, 2023). The first tumor necrosis factor-targeted therapy for RA, infliximab, was approved by the FDA in 1998. Since then, targeted immunotherapies have transformed the treatment of autoimmune diseases, with agents such as adalimumab, pembrolizumab, ibrutinib, and ustekinumab demonstrating substantial clinical efficacy (Jung and Kim, 2022; Klotz et al., 2007). Between 2020 and 2025, the FDA and EMA approved multiple biologics for autoimmune diseases (Supplementary Table S1). For all this progress, autoimmune diseases remain complex disorders influenced by multiple genetic and environmental factors (Díaz-Peña, 2021). Table 3 summarizes engineered therapeutic approaches and their applications in autoimmune diseases.

### 6.5 Neurology

Neurological disorders, such as Alzheimer’s disease, Parkinson’s disease, and neurofibromatosis type 1, affect both the central and peripheral nervous systems (Balakrishnan et al., 2022). Although the underlying causes of many neurological disorders remain unclear, genetic mutations, infections, injuries, and age-related degeneration are known to contribute to disease development (Patel et al., 2016). The complexity of these disorders has limited the effectiveness of conventional therapies: heterogeneity between patients, particularly in Alzheimer’s and Parkinson’s diseases, drives variability in treatment response. Consequently, personalized treatment approaches are essential for improving therapeutic efficacy while minimizing adverse effects (Arnold et al., 2024). For example, some patients with Alzheimer’s disease carry the apolipoprotein E ε4 allele, which influences the response to amyloid-targeting therapies and helps guide treatment selection (Foley and Wilcock, 2024). Similarly, in SMA, genetic and molecular profiling helps identify patients most likely to benefit from exon 7-enhancing therapies such as nusinersen (Dosi and Masson, 2024). Integrating genomic technologies with biomarker analyses profiles patients in molecular detail and supports more precise, effective therapeutic interventions. Table 3 summarizes engineered therapeutic strategies for neurological disorders, and Supplementary Table S1 lists key engineered protein therapeutics, including approved therapies (Knox et al., 2024).

### 6.6 Infectious diseases

Infectious diseases remain a major global health threat, causing millions of deaths annually, particularly in low- and middle-income countries (WHO, n. d.). They arise from pathogenic bacteria, viruses, fungi, and parasites. Although improvements in hygiene, vaccination, and public health infrastructure have reduced the burden of many infections, the overall threat continues to grow because of zoonotic pathogens, increasing globalization, and the rise of antimicrobial resistance (AMR) (Centers for Disease Control and Prevention, 2025). Historically, treatment relied primarily on broad-spectrum antimicrobial agents and public health interventions. However, the limitations of these approaches became evident during global health crises, including the human immunodeficiency virus (HIV) epidemic, the Ebola outbreak, and the coronavirus disease 2019 (COVID-19) pandemic (UNAIDS, 2026), highlighting the need for more precise and adaptive therapeutic strategies.

Precision medicine has increasingly contributed to the management of infectious diseases (Ladner et al., 2019). It has improved diagnostic accuracy, enabled personalized antimicrobial therapy, and enhanced risk assessment for severe clinical outcomes. Precision medicine approaches to infectious disease include mAbs, genome-edited immunotherapies, host-directed biologics, and pathogen-specific diagnostics (U.S. Food and Drug Administration (FDA), 2023a). These technologies modulate immune pathways, disrupt pathogen-host interactions, and directly neutralize infectious agents. For example, long-acting antibodies have shown promise in preventing HIV transmission, whereas combinations of severe acute respiratory syndrome coronavirus 2 (SARS-CoV-2)-neutralizing antibodies reduced hospitalization rates during the COVID-19 pandemic (U.S. Food and Drug Administration (FDA), 2020; Weinreich et al., 2021; Xavier, 2021). Table 3 summarizes engineered therapeutic applications in this area, and several biologics for infectious diseases received FDA and EMA approval between 2020 and 2025 (Supplementary Table S1).

### 6.7 Hematology

Hematology encompasses the study, diagnosis, treatment, and prevention of diseases affecting the blood and hematopoietic system (O’Brien et al., 2021). These disorders include nonmalignant conditions, such as sickle cell disease, hemophilia, and thalassemia, as well as hematologic malignancies, including leukemia, lymphoma, and multiple myeloma. Across both groups, the underlying causes include genetic mutations, epigenetic alterations, immune dysregulation, and environmental factors (O’Brien et al., 2021). Conventional therapeutic approaches often lack precision, leading to variable treatment responses and potential complications among patients. Precision medicine has shifted hematologic treatment from traditional approaches toward mechanism-driven strategies (National Institutes of Health, 2026). For example, next-generation sequencing (NGS) now detects mutations in genes such as *Fms-like tyrosine kinase 3* (*FLT3*), *isocitrate dehydrogenase 1 and 2* (*IDH1/2*), and the *breakpoint cluster region–Abelson* (*BCR-ABL*) fusion gene, thereby guiding targeted therapies for acute leukemia (Association for the Advancement of Blood and Biotherapies, 2025). Clinicians now use biomarkers in both inherited and malignant hematologic disorders to improve diagnostic accuracy, guide treatment selection, and monitor therapeutic response (Santos et al., 2022). Targeted therapies, including tyrosine kinase inhibitors, mAbs, and chimeric antigen receptor T (CAR-T) cell therapies, have significantly improved survival and disease control in patients with hematologic malignancies (Association for the Advancement of Blood and Biotherapies, 2025; June and Sadelain, 2018). CAR-T therapies targeting CD19 and B-cell maturation antigen (BCMA) have demonstrated remarkable efficacy in patients with relapsed or refractory leukemia and multiple myeloma (June and Sadelain, 2018). In addition, gene therapy and genome-editing technologies, such as lentiviral-modified hematopoietic stem cells and clustered regularly interspaced short palindromic repeats (CRISPR)-Cas9, have shown considerable promise in treating inherited blood disorders, including sickle cell disease and β-thalassemia (Frangoul et al., 2021). Advances in recombinant clotting factors and extended half-life products have also transformed hemophilia management by improving treatment efficacy and patient convenience (Bellino et al., 2023). Table 3 summarizes engineered therapeutic approaches for hematologic disorders. Between 2020 and 2025, numerous biologics and gene-based therapies received or are expected to receive regulatory approval, reflecting the ongoing transition toward personalized hematologic care guided by molecular diagnostics and precision therapeutics (Supplementary Table S1).

## 7 Comparative impact of engineered proteins across disease areas and implications for precision medicine

The clinical impact of engineered proteins varies across the therapeutic areas reviewed. Oncology and hematology currently show the greatest clinical translation, with numerous approved mAbs, Fc-engineered variants, bispecific antibodies, and ADCs that are now standard of care (Alcedo Andrade et al., 2024; AlDoughaim et al., 2024; de La Torre and Albericio, 2025; Franchini et al., 2019; Lagassé et al., 2017; Tsimberidou et al., 2020). Similarly, engineered coagulation factors and coagulation factor mimetics have transformed the management of hemophilia and related bleeding disorders by enabling extended dosing intervals and improving prophylactic outcomes (Alcedo Andrade et al., 2024; Franchini et al., 2019). In contrast, few engineered protein therapeutics have been approved for neurological disorders or for many infectious diseases, where persistent challenges remain in BBB penetration, antigenic variability, and long-term safety (de La Torre and Albericio, 2025; Lagassé et al., 2017). Patients with autoimmune diseases and with endocrine and metabolic disorders have benefited substantially from cytokine- and hormone-based biologics; however, important unmet needs remain, including achieving durable remission, minimizing systemic immunosuppression, and improving access to treatment in low- and middle-income countries (de La Torre and Albericio, 2025; Lagassé et al., 2017). Overall, although engineered proteins have reshaped care in certain diseases, other therapeutic areas remain underserved and open to innovation.

Precision medicine has transformed the management of genetic disorders by enabling molecular-level diagnosis and treatment (Awwad et al., 2024). Advances in NGS, bioinformatics, and systems biology let clinicians stratify patients by genetic profile and tailor therapies to specific mutations (Awwad et al., 2024). Protein engineering has evolved from a descriptive discipline into a fundamental component of clinical decision-making. In monogenic disorders, exon skipping, messenger ribonucleic acid delivery, and enzyme engineering restore protein function through targeted molecular modifications (Anastasiou et al., 2025; Anwardeen et al., 2024; Awwad et al., 2024). The resulting engineered proteins are designed to restore normal biological function while minimizing adverse effects. Consequently, comparing the structure and function of engineered proteins with those of their native counterparts indicates therapeutic potential. Such structural matching can reduce adverse immune responses while improving therapeutic efficacy, thereby enabling more informed, evidence-based clinical decisions.

In endocrine and metabolic disorders, dual-agonist peptides for diabetes, gene and cell therapies for autoimmune endocrinopathies, and glycoengineered enzymes for lysosomal storage disorders have demonstrated clinical effectiveness and may alter disease progression (Diseases | GARD, n. d.; “Notable approvals | Drugs | FDA”, n. d.; Anastasiou et al., 2025). These biologics and protein-based therapeutics are central to precision medicine, enabling treatments to be matched to each patient’s molecular profile, with the aim of improving clinical outcomes.

Oncology represents the most advanced precision medicine model, in which engineered protein therapeutics are routinely combined with predictive biomarkers and companion diagnostics to enable patient stratification (AlDoughaim et al., 2024; de La Torre and Albericio, 2025; Jørgensen, 2018; Tsimberidou et al., 2020; Wollenhaupt et al., 2023). Precision oncology integrates genomics, transcriptomics, and proteomics to classify tumors according to their molecular characteristics and enable more effective targeted therapies (Dienstmann et al., 2013). Mechanistically, precision therapeutics target genetic and molecular alterations that disrupt essential cellular processes, such as DNA repair, apoptosis, and cell cycle regulation (Hanahan and Weinberg, 2011). These alterations may be inherited or arise from environmental exposure, aging, or errors during cell replication. This personalized approach is transforming clinical practice by producing more sophisticated prognostic tools, companion diagnostics, and molecularly guided therapies tailored to the biological characteristics of individual tumors (Sharma et al., 2017). Examples include HER2-targeted antibodies and ADCs guided by HER2 expression testing, epidermal growth factor receptor (EGFR)-targeted therapies selected according to tumor mutational status, and immune checkpoint inhibitors, whose use is often guided by programmed death-ligand 1 expression or related biomarkers (Lopatnikova and Sennikov, 2025). The clinical implications of precision medicine in oncology are substantial. Clinicians now select therapeutic strategies on the basis of targetable genetic alterations, such as mutations in EGFR, anaplastic lymphoma kinase, or the breast cancer susceptibility gene. Furthermore, predicting patient responses more accurately increases therapeutic benefit, spares patients ineffective or toxic treatments, reduces healthcare costs, and accelerates drug development through biomarker-guided clinical trial designs (Schwaederle et al., 2015). In addition, machine learning enables complex real-time data analysis and predictive modeling of personalized treatment strategies (Esteva et al., 2019). Liquid biopsies and digital twin models further support adaptive disease management and longitudinal monitoring of treatment responses (Bruynseels et al., 2018; Wan et al., 2017).

Similar principles are emerging in hematology and other fields, where clinicians administer bispecific antibodies and T-cell-engaging constructs to patients whose tumors express specific target antigens. ADCs further illustrate how molecular engineering can deliver cytotoxic payloads selectively to biomarker-defined patient populations (Omer et al., 2023; Wang et al., 2025). In hematologic disorders, engineered therapeutic approaches include CAR-T cells and protein-based T-cell engagers such as BiTEs, which recognize specific target antigens, including CD19 or BCMA (U.S. Food and Drug Administration (FDA), 2026c; June and Sadelain, 2018; Tapia-Galisteo et al., 2023). Clinicians increasingly use pharmacogenomics to predict drug metabolism and toxicity, improving treatment safety and efficacy. Together, these technologies enable them to tailor therapeutic interventions according to specific genetic mutations, gene expression signatures, and immunophenotypic profiles (Association for the Advancement of Blood and Biotherapies, 2025; National Institutes of Health, 2026). In this context, engineered proteins, including bispecific antibodies, ADCs, and extended half-life coagulation factors, expand the available therapeutic options and advance precision medicine by integrating target selection, patient stratification, and individualized dosing strategies (Lu et al., 2020; Lu et al., 2025).

Outside oncology, however, robust biomarkers and companion diagnostics remain scarce. Closing this gap is a major opportunity to broaden precision protein therapeutics across the other disease areas discussed in this review. In autoimmune diseases, recombinant protein biologics, such as mAbs, fusion proteins, and engineered cytokines, are designed to selectively modulate key regulatory pathways involved in disease pathogenesis rather than broadly suppress the immune system (Sadelain et al., 2013). Because these diseases are complex, clinical decision-making must move beyond empirical treatment toward precision-based interventions. Genetic predisposition, environmental exposures, and interpatient variability contribute to unpredictable therapeutic responses and present significant challenges for drug development. Consequently, precision medicine could improve the management of autoimmune diseases by tailoring treatment strategies to individual patient characteristics, disease subtypes, and genetic profiles. Advanced diagnostic tools help predict treatment response, monitor disease activity, and identify novel therapeutic targets (Díaz-Peña, 2021).

Neurological disorders are similarly complex and require therapeutic proteins designed with improved functional properties to cross the BBB and inhibit pathological processes such as toxic protein aggregation (Pena et al., 2020). Receptor-mediated transcytosis actively carries therapeutic proteins across the BBB into the central nervous system (Pena et al., 2020).

In infectious diseases, precision medicine integrates host and pathogen genomic data, immune profiling, and real-time surveillance (World Health Organization, 2025). This approach enables clinicians to tailor interventions to individual patient characteristics, including genetic susceptibility, immune status, and microbiome composition (World Health Organization, 2025; World Health Organization, 2026b). High-throughput genome sequencing and bioinformatics now rapidly identify infectious pathogens, characterize AMR mechanisms, and monitor disease transmission (van Belkum et al., 2019; World Health Organization, 2026a). Precision medicine also supports personalized therapeutic approaches, including biologics that target specific pathogens or modulate host immune responses (Equils et al., 2023). At the molecular level, engineered therapeutic proteins disrupt pathogen-host interaction pathways, blocking pathogen entry into host cells and neutralizing microbial toxins (Walker and Burton, 2018).

## 8 Challenges and future directions

Therapeutic protein production is a cornerstone of precision medicine, biotechnology, and therapeutic development, supplying proteins for research, diagnostics, and clinical applications. Despite its numerous advantages, this process presents several technical and biological challenges that can limit its efficiency, functionality, and scalability. The primary challenges are related to protein solubility, folding, and PTMs. These factors indirectly affect manufacturers by increasing production complexity and costs and by limiting the availability of therapeutic proteins (Izutsu, 2024). Immunogenicity is another major concern, as engineered proteins may induce immune responses through the formation of anti-drug antibodies. Such immune responses can reduce therapeutic efficacy or cause adverse effects. Immunogenicity may arise from preexisting antiviral antibodies, rapid innate immune responses, or adaptive immune responses directed against viral vectors rather than tumor-specific antigens. In addition, therapeutic proteins can interact with receptors expressed on antigen-presenting cells (APCs), triggering adaptive immune responses. In another pathway, immature dendritic cells can internalize therapeutic proteins through phagocytosis and process them via the major histocompatibility complex class II pathway (Baker et al., 2010). Reducing immunogenicity remains one of the principal challenges in therapeutic protein development. Future research should prioritize integrating *in silico*, *in vitro*, and *in vivo* approaches to improve the prediction of immunogenicity (De Groot et al., 2008). In addition, how these therapies influence immune responses in individual patients or small patient cohorts remains poorly understood. Concerns regarding immunogenicity remain significant among both clinicians and patients in the context of precision medicine.

Another important challenge involves regulatory pathways and their impact on manufacturers, particularly for protein-based therapeutics. Regulatory approval processes are complex and are often not fully aligned with the unique characteristics of precision medicine, leading to delays and uncertainty in bringing these therapies to market (Gutierrez et al., 2020). We agree with Gutierrez et al. (2020) that Chemistry, Manufacturing, and Controls (CMC) data should be automated and made available through a centralized cloud-based platform accessible to regulatory agencies worldwide. A shared CMC platform could reduce submission costs, facilitate simultaneous global submissions, and shorten regulatory review timelines. Consequently, this approach could accelerate the adoption of new biotechnologies by manufacturers and improve patient access to innovative therapies. Nevertheless, manufacturers must still meet stringent regulatory requirements when implementing new technologies to ensure compliance with quality standards. In addition, purification, downstream processing, and viral inactivation procedures remain major cost drivers in therapeutic protein manufacturing (Sifniotis et al., 2019).

Ethical considerations pose another major challenge, particularly unequal access to therapeutic protein treatments across different populations, an inequity that may contribute to substantial healthcare disparities. Access to CAR-T-cell therapy remains limited for underserved and minority populations (Odstrcil et al., 2024). These groups often experience barriers to obtaining adequate health insurance, which reduce their access to advanced cellular therapies when needed. In addition, CAR-T-cell manufacturing requires several weeks to produce the final therapeutic product, creating further logistical challenges (Nze and Flowers, 2023). Another widely debated issue is the FDA approval of Aduhelm (aducanumab; Biogen Inc., Cambridge, Massachusetts) despite limited evidence demonstrating improvements in cognitive or functional outcomes in Alzheimer’s disease (Glymour et al., 2022). Privacy of genomic data also represents a critical ethical concern. Individuals may fear that their genomic information could be misused or lead to discrimination, particularly when combined with algorithm-based decision-making systems that may introduce institutional or societal bias. Furthermore, genomic information obtained from one individual may inadvertently reveal sensitive information about biologically related family members because genetic information is inherently shared among relatives (ICO, 2024).

Implementing precision medicine also raises important challenges related to genetic diversity. Precision medicine relies heavily on genetic studies, which are often conducted in relatively small populations and may not adequately represent all racial or ethnic groups. In addition, applying pharmacogenetics requires extensive effort to obtain highly accurate genetic information. Although pharmacogenetic testing can help identify individuals who are more likely to respond to specific therapies, several challenges remain. Limited understanding of pharmacogenetic variation may contribute to suboptimal healthcare for certain populations. Another major ethical concern involves patient privacy and informed consent. Protecting patient data and ensuring that patients fully understand how their genetic information will be collected, stored, and used remain fundamental ethical requirements (Dickens et al., 2021). Addressing these scientific, regulatory, and ethical challenges will require coordinated efforts from researchers, regulatory agencies, healthcare providers, and policymakers. Ensuring equitable global access to these emerging technologies is essential for realizing the full potential of precision medicine (Mentis et al., 2018; Shih et al., 2022).

Advances in genomics, proteomics, and metabolomics, collectively referred to as multi-omics, are transforming precision medicine by providing a more comprehensive understanding of disease mechanisms and pathophysiology. Integrating these datasets supports the development of increasingly precise diagnostic and therapeutic strategies (Mani et al., 2025; Molla and Bitew, 2024). Another important development is the application of AI and machine learning to therapeutic protein design. Investment in AI has enabled rapid and accurate prediction of protein structures, optimization of drug candidates, and interpretation of complex biological datasets. AlphaFold, for example, has substantially advanced protein structure prediction, representing a major breakthrough in drug discovery (Branca, 2024; Filipp, 2019). In addition, proteolysis-targeting chimeras (PROTACs) have emerged as an innovative strategy for targeted protein degradation. Personalized cancer vaccines are also being developed to target patient-specific tumor antigens, thereby improving the specificity and efficacy of cancer immunotherapy (Braselmann et al., 2020).

Although these challenges are well recognized in therapeutic protein development, their implications for precision medicine are even more significant. For example, immunogenicity undermines the fundamental principle of patient-specific treatment by introducing unpredictable variability in drug exposure and clinical response. This variability reduces the reliability of biomarkers and genetic profiles for patient stratification, particularly when anti-drug antibody formation differs among racial, genetic, or disease-specific subgroups. Regulatory challenges are also especially relevant to precision medicine because biomarker-guided therapies require concurrent validation of companion diagnostics and subgroup-specific dosing strategies, together with evidence demonstrating consistent performance across genetically diverse populations. Addressing the limited availability of validated biomarkers and companion diagnostics outside oncology will require the development and qualification of disease-specific predictive biomarkers and clearer regulatory pathways for their clinical implementation. In autoimmune diseases, for example, biomarker qualification could support patient stratification and identify individuals most likely to benefit from targeted protein therapeutics. Ethical issues, including unequal access to genomic testing, disparities in insurance coverage for advanced biologics and cellular therapies, and concerns regarding the ownership and use of genomic data, further limit the practical implementation of precision medicine, even when effective engineered protein therapeutics are available. Collectively, these challenges demonstrate that the barriers extend beyond scientific and manufacturing considerations and directly influence who can benefit from precision protein therapeutics, how reliably treatments can be personalized, and how rapidly these therapies can reach patients.

Despite the broad scope of this review, several limitations should be acknowledged. Because protein engineering and precision medicine are evolving rapidly, some emerging technologies and recent regulatory approvals may not have been captured at the time of writing. In addition, the cross-disease comparison of clinical translation presented in this review is based primarily on patterns and numbers of regulatory approvals rather than comparative clinical outcome data. Accordingly, it should be interpreted as reflecting regulatory activity and the extent of therapeutic translation across disease areas rather than differences in clinical benefit or patient outcomes. Moreover, because peer-reviewed evidence remains limited in certain therapeutic areas, such as neurology and rare genetic disorders, the depth of discussion in these sections is constrained by the availability and quality of published studies. Furthermore, this review did not employ a systematic literature search but instead focused on representative and high-impact publications, which may have introduced selection bias. Finally, the current evidence base relies heavily on early-stage, preclinical, or small-cohort studies, limiting the ability to draw definitive conclusions regarding long-term safety, real-world effectiveness, and applicability across diverse patient populations.

## 9 Conclusion

This review highlights recent advances in protein engineering and clinical progress across major therapeutic areas, providing an integrated perspective on how engineered proteins are shaping the development of precision medicine. By compiling FDA- and EMA-approved engineered protein therapeutics from 2020 to 2025, this review documents the accelerating regulatory momentum in the field and underscores the rapid translation of novel protein modalities into clinical practice. Despite these advances, several challenges remain, including balancing molecular specificity with manufacturing scalability and ensuring ethical, equitable access to biomarker-driven therapies. Addressing these challenges is critical for the widespread implementation of precision medicine on a global scale.

The future of precision medicine is expected to be driven by emerging technologies, including AI-driven protein design and machine learning. These advances are reshaping therapeutic development by improving the accuracy of protein structure prediction and enabling the design of proteins with tailored functional properties. In addition, targeted protein degradation platforms, such as PROTACs, and the development of highly personalized cancer vaccines represent promising opportunities for next-generation precision therapeutics. Collectively, these innovations should accelerate the use of engineered proteins in precision medicine, enabling more precise, patient-specific therapies and increasingly tailored, data-driven treatment strategies.
