# Supplementary material for: Advances and applications of engineered protein therapeutics and their role in precision medicine
Source: Front Bioeng Biotechnol. 2026 Oct 5;14:1857362. doi: 10.3389/fbioe.2026.1857362 (PMC13678489; doi:10.3389/fbioe.2026.1857362)
Supplement: Supplementary file 1 [file Table1.docx]

Supplementary Material

# 1 Table S1. Selected biologics approved between 2020 and 2025

| **Oncology** (European Medicines Agency (EMA), n.d.; FDA, n.d.) | | | | |
| --- | --- | --- | --- | --- |
| **Generic name** | **Target/mechanism** | **Indicate/condition** | **Year of approval** | **Company/developer** |
| Lisocabtagene maraleucel | Autologous T cells engineered to target CD19 antigen. | Relapsed/refractory large B-cell lymphoma | FDA (n.d.), EMA (n.d.) | Juno Therapeutics |
| Brexucabtagene autoleucel | Autologous T cells engineered to target CD19 antigen. | Relapsed/refractory mantle cell lymphoma | FDA (n.d.), EMA (n.d.) | Kite pharma |
| Ciltacabtagene autoleucel | Autologous T cells engineered to target BCMA antigen. | Relapsed/refractory multiple myeloma | FDA (n.d.), EMA (n.d.) | Jansson and Legend Biotech |
| Lifileucel | Autologous tumor infiltrating lymphocytes expanded ex vivo to target tumor. | Unresectable/metastatic melanoma | FDA (n.d.), EMA (n.d.) | Lovance Biotherapeutics |
| Mosunetuzumab | Dual ScFv linking CD3 on T cells and tumor antigen to induce cytotoxicity. | Relapsed/refractory follicular lymphoma | FDA (n.d.), EMA (n.d.) | Genentech/Roche |
| Teclistamab (FDA n.d.) | Dual ScFv targeting BCMA and CD3. | Relapsed/refractory multiple myeloma | FDA (n.d.), EMA (n.d.) | Janssen Pharmaceuticals |
| Tebentafusp | Dual specificity peptide‑HLA×CD3 to redirect T cells. | Unresectable/metastatic uveal melanoma | FDA (n.d.), EMA (n.d.) | Immunocore |
| Epcoritamab | Dual ScFv targeting CD20 and CD3. | Relapsed/refractory follicular lymphoma | FDA (n.d.) | Genmab & AbbVie |
| Glofitamab | Dual ScFv targeting CD20 and CD3. | Relapsed/refractory DLBCL | FDA (n.d.), EMA (n.d.) | Roche |
| Talquetamab | Dual ScFv targeting GPRC5D and CD3. | Relapsed/refractory multiple myeloma | FDA (n.d.), EMA (n.d.) | Janssen Pharmaceuticals |
| Nivolumab+relatlimab (FDA n.d.) | Dual checkpoint inhibition (PD‑1 + LAG‑3) to reverse T-cell exhaustion. | Unresectable/metastatic melanoma | FDA (n.d.), EMA (n.d.) | Bristol-Myers Squibb |
| Dostarlimab | Humanized anti‑PD‑1 monoclonal antibody blocking T cell inhibition. | dMMR endometrial cancer | FDA (n.d.), EMA (n.d.) | GSK |
| Atezolizumab + bevacizumab | Anti‑PD‑L1 plus anti‑VEGF to enhance immune/anti‑angiogenic responses. | Unresectable/metastatic HCC | FDA (n.d.), EMA (n.d.) | Roche |
| Amivantamab | Dual specificity EGFR×MET targeting EGFR exon 20‑insertion NSCLC. | EGFR exon20‑insertion–mutant NSCLC | FDA (n.d.), EMA (n.d.) | Janssen Biotech (J&J) |
| Sacituzumab govitecan | Anti‑TROP‑2 antibody linked to cytotoxic payload. | Relapsed/refractory TNBC | FDA (n.d.), EMA (n.d.) | Gilead Sciences |
| Belantamab mafodotin | Anti‑BCMA antibody linked to microtubule toxin. | Relapsed/refractory multiple myeloma | FDA (n.d.) | GSK |
| Loncastuximab tesirine | Anti‑CD19 antibody linked to pyrrolobenzodiazepine toxin. | Relapsed/refractory large B‑cell lymphoma | FDA (n.d.), EMA (n.d.) | ADC therapeutics |
| Tisotumab vedotin | Anti‑tissue factor antibody conjugated with cytotoxic payload. | Recurrent/metastatic cervical cancer | FDA (n.d.), EMA (n.d.) | Pfizer |
| Trastuzumab deruxtecan | Anti‑HER2 antibody linked to topoisomerase inhibitor. | HER2‑mutant NSCLC | FDA (n.d.) | Daiichi Sankyo & AstraZeneca |
| Mirvetuximab soravtansine (FDA n.d.) | Anti‑folate receptor α antibody linked to maytansinoid. | Platinum‑resistant ovarian cancer | FDA (n.d.), EMA (n.d.) | AbbVie |
| Isatuximab | Anti‑CD38 antibody promoting immune‑mediated cytotoxicity. | Relapsed/refractory multiple myeloma | FDA (n.d.), EMA (n.d.) | Sanofi |
| Tafasitamab | Fc‑enhanced anti‑CD19 antibody to improve ADCC. | Relapsed/refractory DLBCL | FDA (n.d.) | MorphoSys & Incyte corporation |
| Naxitamab | Anti‑GD2 antibody targeting neuroblastoma cells. | Refractory/relapsed high‑risk neuroblastoma | FDA (n.d.) | Y-mAbs Therapeutics |
| Margetuximab | Fc‑engineered anti‑HER2 antibody with enhanced ADCC. | HER2⁺ metastatic breast cancer | FDA (n.d.), EMA (n.d.) | MacroGenics, Inc |
| Tremelimumab | Anti‑CTLA‑4 antibody used in combo with anti‑PD‑L1. | Unresectable hepatocellular carcinoma | FDA (n.d.) | AstraZeneca |
| Elranatamab | Dual ScFv targeting BCMA and CD3. | Relapsed/refractory multiple myeloma | FDA (n.d.) | Pfizer |
| Retifanlimab | Anti‑PD‑1 antibody targeting PD‑1. | Metastatic/recurrent Merkel cell carcinoma | FDA (n.d.), EMA (n.d.) | Incyte corporation |
| Afamitresgene autoleucel | Autologous T cells engineered to express TCR targeting MAGE‑A4. | Advanced synovial sarcoma | FDA (n.d.) | Adaptimmune Therapeutics |
| **Genetic disorders** (Knox et al. 2024 | | | | |
| Atidarsagene autotemcel | Lentiviral-modified autologous HSCs | Metachromatic leukodystrophy (MLD) | 2020 (EMA), 2024 (FDA) | Orchard Therapeutics |
| Strimvelis | Autologous gene-corrected HSCs | ADA-SCID | 2016 (EMA) | Orchard Therapeutics |
| Revakinagene taroretcel | Encapsulated cell-based gene therapy | Macular telangiectasia type 2 | 2025 (FDA) | Nanoscope Therapeutics |
| Avalglucosidase alfa | Recombinant human GAA enzyme | Late-onset Pompe disease | 2021 (FDA), | Sanofi |
|  |  |  | 2022 (EMA) |  |
| Givinostat | HDAC inhibitor | Duchenne muscular dystrophy | 2024 (FDA) | Italfarmaco |
| Exagamglogene autotemcel | CRISPR-Cas9 gene editing | Sickle cell disease | 2023 (FDA) | Vertex/CRISPR Therapeutics |
| Onasemnogene abeparvovec | AAV9 vector-based gene therapy | Spinal muscular atrophy (SMA) | 2019 (FDA), | Novartis (AveXis) |
|  |  |  | 2020 (EMA) |  |
| Lovotibeglogene autotemcel | Lentiviral gene therapy | Sickle cell disease | 2023 (FDA) | bluebird bio |
| Delandistrogene Moxeparvovec | AAV-based gene therapy | Duchenne muscular dystrophy | 2023 (FDA) | Sarepta Therapeutics |
| Fidanacogene elaparvovec | AAV-based gene therapy | Hemophilia B | 2024 (FDA) | Pfizer |
| Etranacogene dezaparvovec | AAV-based gene therapy | Hemophilia B | 2022 (FDA) | uniQure/CSL Behring |
| Voretigene neparvovec | AAV-based gene therapy | Leber congenita amaurosis (RPE65 mutation) | 2017 (FDA), | Spark Therapeutics |
|  |  |  | 2018 (EMA) |  |
| Eladocagene exuparvovec | AAV2-based gene therapy | AADC deficiency | 2024 (FDA) | PTC Therapeutics |
| Beremagene geperpavec | HSV-1 vector-based gene therapy | Dystrophic epidermolysis bullosa | 2023 (FDA) | Krystal Biotech |
| Casimersen | Antisense oligonucleotide (exon 45 skipping) | DMD (exon 45 amenable) | 2021 (FDA) | Sarepta Therapeutics |
| Fitusiran | siRNA targeting antithrombin | Hemophilia A and B (± inhibitors) | 2025 (FDA) | Alnylam/Sanofi |
| Viltolarsen | Antisense oligonucleotide (exon 53 skipping) | DMD (exon 53 amenable) | 2020 (FDA) | NS Pharma (Nippon Shinyaku) |
| Golodirsen | Antisense oligonucleotide (exon 53 skipping) | DMD (exon 53 amenable) | 2019 (FDA) | Sarepta Therapeutics |
| Vamorolone | Modified steroid with reduced side effects | Duchenne Muscular Dystrophy | 2023 (FDA) | Santhera Pharmaceuticals |
| **Endocrinology and metabolic disorders (Knox et al. 2024)** | | | | |
| Eladocagene exuparvovec | In vivo gene therapy (DDC gene) | AADC deficiency | 2022 (EMA) | PTC Therapeutics |
| Donislecel | Allogeneic islet cell therapy | Type 1 diabetes | 2023 (FDA) | CellTrans |
| Somatrogon | GH fusion protein (extended half-life) | Pediatric GH deficiency | 2023 (FDA), | Pfizer/OPKO Health |
|  |  |  | 2022 (EMA) |  |
| Atidarsagene autotemcel | Ex vivo gene therapy (ARSA gene) | Metachromatic leukodystrophy (MLD) | 2020 (EMA), 2024 (FDA) | Orchard Therapeutics |
| Elivaldogene autotemcel | Ex vivo gene therapy (ABCD1 gene) | Cerebral adrenoleukodystrophy (CALD) | 2021 (EMA), 2023 (FDA) | bluebird bio |
| Somapacitan | Long-acting GH analog | Adult growth hormone deficiency | 2020 (FDA) | Novo Nordisk |
| Lonapegsomatropin | Long-acting GH prodrug (TransCon) | Pediatric GH deficiency | 2021 (FDA) | Ascendis Pharma |
| Dasiglucagon | Glucagon receptor agonist | Severe hypoglycemia | 2021 (FDA) | Zealand Pharma |
| Teplizumab | Anti-CD3 monoclonal antibody | Type 1 diabetes (delayed onset) | 2022 (FDA) | Provention Bio/Sanofi |
| Teprotumumab | IGF-1R antagonist monoclonal antibody | Thyroid eye disease | 2020 (FDA) | Horizon Therapeutics |
| Evinacumab | ANGPTL3 monoclonal antibody | Homozygous familial hypercholesterolemia (HoFH) | 2021 (FDA) | Regeneron |
| Pegunigalsidase alfa | PEGylated α-galactosidase A (ERT) | Fabry disease | 2023 (FDA), | Chiesi/Protalix |
|  |  |  | 2022 (EMA) |  |
| Tirzepatide | Dual GIP/GLP-1 receptor agonist | Type 2 diabetes | 2022 (FDA) | Eli Lilly |
| Semaglutide 2.4mg | GLP-1 receptor agonist | Obesity (chronic weight management) | 2021 (FDA) | Novo Nordisk |
| Setmelanotide | Melanocortin-4 receptor agonist | Genetic obesity (POMC, LEPR deficiencies) | 2020 (FDA), | Rhythm Pharmaceuticals |
|  |  |  | 2021 (EMA) |  |
| Avalglucosidase alfa | Acid α-glucosidase (ERT) | Pompe disease | 2021 (FDA) | Sanofi |
| Olipudase alfa | Sphingomyelinase (ERT) | Acid sphingomyelinase deficiency | 2022 (FDA) | Sanofi |
| Inclisiran | siRNA targeting PCSK9 mRNA | Hypercholesterolemia | 2021 (FDA), | Alnylam/Novartis |
|  |  |  | 2020 (EMA) |  |
| Givosiran | siRNA targeting ALAS1 | Acute hepatic porphyria | 2019 (FDA) | Alnylam Pharmaceuticals |
| Lumasiran | siRNA targeting HAO1 | Primary hyperoxaluria type 1 | 2020 (EMA), 2021 (FDA) | Alnylam Pharmaceuticals |
| **Autoimmune disorders** (Knox et al. 2024) | | | | |
| Cytokine Inhibitors | | | | |
| Nemolizumab | IL-31 | Prurigo nodularis and atopic dermatitis | 2022, 2024 - Japan, FDA | Nemluvio/Chugai Pharmaceutical Co., Ltd., Galderma |
| Anifrolumab | Type I interferon receptor | Systemic lupus erythematosus (SLE) | 2021 - FDA | Saphnelo/Medarex |
|  | | | | |
| Spesolimab | IL-36 | Pustular psoriasis flares in adults | 2022 - FDA | Spevigo/Boehringer Ingelheim |
| Bimekizumab | IL-17A, IL-17F, IL-17AF | Psoriasis | 2023 - EMA, FDA | Bimzelx/Ucb |
| Satralizumab | IL-6 | Neuromyelitis optical spectrum disorder | 2020 - Canada, Japan, EMA and FDA | Enspryng/Chugai Pharmaceutical and Roche |
| Mirikizumab | IL-23 | Ulcerative colitis | 2023 - Canada, Japan, EMA and FDA | Omvoh/Eli Lilly and Company |
| **Immune cell inhibitors** | | | | |
| Inebilizumab | CD19 on B-cells | Neuromyelitis optical spectrum disorder | 2020 - FDA | Uplizna/Horizon Therapeutics USA, Inc., Amgen |
|  | | | | |
| Ublituximab | CD20 on B, T-cells | Relapsing forms of multiple sclerosis | 2022, 2023 - FDA, EMA | Briumvi/TG Therapeutics |
| Ofatumumab | CD20 on B cells |  | 2020 - EMA, FDA | Arzerra, Kesimpta/Genmab |
| Teplizumab | CD3 on T-cells | Type 1 diabetes | 2022 - FDA | Tzield/MacroGenics and Provention Bio |
| **Receptor inhibitors** | | | | |
| Rozanolixizumab | Neonatal Fc receptor | Myasthenia gravis | 2020, 2024 - FDA, EMA, Japan | Rystiggo/UCB Pharma |
| Efgartigimod alfa |  |  | 2021 - FDA, EMA | Vyvgart, Vyvgart Hytrulo/Argenx |
| Nipocalimab |  |  | 2025 - FDA | Imaavy/anssen Research & Development, LLC |
| Teprotumumab | Insulin-like growth factor-1 receptor | Thyroid eye disease. | 2020 - FDA | Tepezza/Horizon Therapeutics |
| **Complement inhibitors** | | | | |
| Sutimlimab | C1s | Cold agglutinin disease (CAD) | 2022 - FDA, EMA | Enjaymo/Sanofi |
| **Neurological disorders** (Knox et al. 2024) | | | | |
| **Amyloid beta inhibitors** | | | | |
| Aducanumab | Amyloid-beta plaques | Alzheimer’s disease | 2021 - FDA | Aduhelm/Biogen |
| Lecanemab |  |  | 2023 - FDA | Leqembi/Eisai and Biogen |
| Donanemab |  |  | 2024 - FDA | Kisunla/Eli Lilly and Company |
| **Complement inhibitors** | | | | |
| Ravulizumab |  | Paroxysmal nocturnal hemoglobinuria, atypical hemolytic uremic syndrome, myasthenia gravis | 2021 - FDA | Ultomiris/Alexion Pharmaceuticals |
|  | Complement C5 |  |  |  |
| Zilucoplan |  | Myasthenia gravis in anti-acetylcholine receptor (AChR) antibody-positive adults | 2023 - FDA, EMA | Zilbrysq/Ra Pharmaceuticals |
| **CGRP inhibitors** | | | | |
| Eptinezumab | human calcitonin gene-related peptide (CGRP) | migraine | 2020 - FDA | Vyepti/Lundbeck Seattle BioPharmaceuticals, Inc |
| **Nucleic acid-based therapies** | | | | |
| Delandistrogene moxeparvovec |  |  | 2023 - FDA | Elevidys/Sarepta Therapeutics in collaboration with Roche |
|  |  |  |  |  |
| Viltolarsen | Duchenne muscular dystrophy (DMD) gene | DMD | 2020 - FDA | Viltepso/NS Pharma, Inc., a wholly-owned subsidiary of Nippon Shinyaku, in collaboration with the National Center of Neurology and Psychiatry (NCNP) |
| Casimersen |  |  | 2021 - FDA | Amondys 45/Sarepta Therapeutics |
| Tofersen | Mutated SOD1 gene | Amyotrophic lateral sclerosis | 2023, 2024 - FDA, EMA | Qalsody/Ionis Pharmaceuticals |
| **Infectious diseases (Knox et al. 2024)** | | | | |
| Generic name | Target/mechanism | Indicate/condition | Year of Approval | Company/Developer |
| Atoltivimab + Maftivimab + Odesivimab-ebgn | Ebola virus glycoprotein (neutralization) | Zaire ebolavirus infection | FDA (n.d.) | Regeneron |
| Ansuvimab-zykl | Ebola virus glycoprotein | Zaire ebolavirus infection | FDA (n.d.) | Ridgeback Biotherapeutics |
| Obiltoxaximab | Bacillus anthracis protective antigen | Inhalational anthrax | FDA (initial: 2016), reaffirmed in 2020 | Elusys Therapeutics |
| BNT162b2 (tozinameran) | SARS-CoV-2 spike protein mRNA | COVID-19 prevention | FDA (n.d.), EMA (n.d.) | Pfizer/BioNTech |
| Meningococcal groups A, C, W, Y conjugate vaccine | Polysaccharide-protein conjugate | Meningococcal disease prevention | FDA/EMA (various formulations) | Sanofi |
| ChAdOx1 nCoV-19 (AZD1222) | SARS-CoV-2 spike protein (adenoviral vector) | COVID-19 prevention | EMA (n.d.), Not FDA-approved | AstraZeneca/Oxford |
| Ad26.COV2.S (recombinant) | SARS-CoV-2 spike protein (adenoviral vector) | COVID-19 prevention | FDA (n.d.), EMA (n.d.) | Janssen (J&J) |
| mRNA-1273 (elasomeran) | SARS-CoV-2 spike protein mRNA | COVID-19 prevention | FDA (n.d.), EMA (n.d.) | Moderna |
| Regdanvimab | SARS-CoV-2 spike protein | COVID-19 treatment | EMA only (2021) – Not FDA-approved | Celltrion |
| Casirivimab + Imdevimab | SARS-CoV-2 spike protein | COVID-19 treatment | FDA EUA (2020, revoked 2022), EMA (conditional 2021) | Regeneron |
| Sotrovimab | SARS-CoV-2 spike protein | COVID-19 treatment | FDA EUA (2021, withdrawn), EMA (n.d.) | GlaxoSmithKline/Vir |
| Tick-borne encephalitis vaccine (inactivated) | Inactivated virus | TBE prevention | EMA (approved); Not FDA-approved | Pfizer |
| 3-antigen hepatitis B vaccine (recombinant, adsorbed) | HBV S, pre-S1, and pre-S2 antigens | Hepatitis B prevention | FDA (n.d.), EMA (n.d.) | VBI Vaccines |
| NVX-CoV2373 | Recombinant SARS-CoV-2 spike protein + Matrix-M adjuvant | COVID-19 prevention | FDA (n.d.), EMA (n.d.) | Novavax |
| Tixagevimab + Cilgavimab | SARS-CoV-2 spike protein | COVID-19 prophylaxis (pre-exposure) | FDA EMA (n.d.), EMA (n.d.) | AstraZeneca |
| TAK-003 | Live-attenuated dengue virus (4 serotypes) | Dengue fever prevention | EMA (n.d.) and FDA (n.d.) | Takeda |
| Nirsevimab-alip | RSV fusion protein (neutralizing antibody) | RSV prophylaxis in infants | EMA (n.d.) and FDA (n.d.) | Sanofi/AstraZeneca |
| RSV prefusion F vaccine (adjuvanted) | RSV F protein (adjuvanted) | RSV prevention | FDA (n.d.), EMA (n.d.) | GSK |
| RSV prefusion F vaccine (bivalent, unadjuvanted) | RSV F protein (bivalent) | RSV prevention | FDA (n.d.), EMA (n.d.) | Pfizer |
| MenACWY-TT/MenB-FHbp | Pentavalent meningococcal vaccine | Meningococcal disease prevention | FDA (n.d.) | Pfizer |
| Fecal microbiota, live – jslm | Restoration of gut microbiota | Recurrent *Clostridium difficile* infection | FDA (n.d.), EMA (n.d.) | Ferring Pharmaceuticals |
| Purified firmicutes spores (oral microbiota capsules) | Firmicutes bacterial spores | Recurrent *C. difficile* infection | FDA (n.d.), Not EMA-approved yet | Seres Therapeutics |
| MenABCWY | Pentavalent meningococcal vaccine | Meningococcal disease prevention | FDA (n.d.), EMA (n.d.) | GSK |
| **Hematology (Knox et al. 2024)** | | | | |
| Generic name | Target/mechanism | Indicate/condition | Approval (Year & Authority) | Company/Developer |
| Betibeglogene autotemcel | Gene addition (beta-globin) | Beta-thalassemia | 2022 - FDA | Bluebird Bio |
| Lovotibeglogene autotemcel | Gene editing (HbF reactivation via BCL11A) | Sickle cell disease | 2023 - FDA | Vertex/CRISPR Therapeutics |
| Exagamglogene autotemcel | Gene editing (CRISPR/Cas9 BCL11A) | Sickle cell disease, Beta-thalassemia | 2023 - EMA | Vertex/CRISPR Therapeutics |
| Valoctocogene roxaparvovec-rvox | AAV5-mediated factor VIII gene therapy | Hemophilia A | 2023 - FDA & EMA | BioMarin |
| Etranacogene dezaparvovec-drlb | AAV5-mediated factor IX gene therapy | Hemophilia B | 2022 - FDA & EMA | uniQure/CSL Behring |
| Brexucabtagene autoleucel | CD19-directed CAR-T | Mantle cell lymphoma | 2020 - FDA & EMA | Kite Pharma (Gilead) |
| Lisocabtagene maraleucel | CD19-directed CAR-T | Large B-cell lymphoma | 2021 - FDA & EMA | Bristol Myers Squibb |
| Idecabtagene vicleucel | BCMA-directed CAR-T | Multiple myeloma | 2021 - FDA & EMA | Bristol Myers Squibb/bluebird bio |
| Ciltacabtagene autoleucel | BCMA-directed CAR-T | Multiple myeloma | 2022 - FDA & EMA | Janssen/Legend Biotech |
| Isatuximab-irfc | CD38 monoclonal antibody | Multiple myeloma | 2020 - FDA & EMA | Sanofi |
| Tafasitamab-cxix | CD19 monoclonal antibody | Diffuse large B-cell lymphoma | 2020 - FDA | Incyte/MorphoSys |
| Mosunetuzumab-axgb | CD20/CD3 bispecific antibody | Follicular lymphoma | 2022 - FDA & EMA | Genentech (Roche) |
| Epcoritamab-bysp | CD20/CD3 bispecific antibody | DLBCL and follicular lymphoma | 2023 - FDA & EMA | Genmab/AbbVie |
| Glofitamab-gxbm | CD20/CD3 bispecific antibody | B-cell lymphomas | 2023 - FDA & EMA | Genentech (Roche) |
| Teclistamab-cqyv | BCMA/CD3 bispecific antibody | Multiple myeloma | 2022 - FDA & EMA | Janssen |
| Talquetamab-tgvs | GPRC5D/CD3 bispecific antibody | Multiple myeloma | 2023 - FDA | Janssen |
| Elranatamab-bcmm | BCMA/CD3 bispecific antibody | Multiple myeloma | 2023 - FDA | Pfizer |
| Belantamab mafodotin-blmf | BCMA antibody-drug conjugate | Multiple myeloma | 2020 - FDA (withdrawn), EMA | GSK |
| Loncastuximab tesirine-lpyl | CD19 antibody-drug conjugate | Large B-cell lymphoma | 2021 - FDA | ADC Therapeutics |
| Sutimlimab-jome | C1s complement inhibitor | Cold agglutinin disease | 2022 - FDA | Sanofi |
| Concizumab-mtci | Anti-TFPI monoclonal antibody | Hemophilia A and B | 2023 - EMA | Novo Nordisk |
| Crizanlizumab-tmca | P-selectin inhibitor | Sickle cell disease (pain crises) | 2019 (initial), reaffirmed 2020 - FDA | Novartis |
| Pegcetacoplan | C3 complement inhibitor | Paroxysmal nocturnal hemoglobinuria (PNH) | 2021 - FDA & EMA | Apellis |

EMA, European Medicines Agency; FDA, Food and Drug Administration; ERT, enzyme replacement therapy; AAV, adeno-associated virus; HSC, hematopoietic stem cell; GH, growth hormone.

# 3 References

European Medicines Agency (EMA). (n.d.). Cancer medicines. <https://www.ema.europa.eu/en/human-regulatory-overview/research-development/cancer-medicines#in-this-section-69509>.

FDA. (n.d.). Oncology (cancer)/hematologic malignancies approval notifications. <https://www.fda.gov/drugs/resources-information-approved-drugs/oncology-cancerhematologic-malignancies-approval-notifications>.

Knox, C., Wilson, M., Klinger, C. M., Franklin, M., Oler, E., Wilson, A. et al. (2024). DrugBank 6.0: The DrugBank knowledgebase for 2024. Nucleic Acids Res. 52, D1265–D1275. doi: [10.1093/NAR/GKAD976](https://doi.org/10.1093/nar/gkad976).
